# Endodontic education - present status and future directions

**DOI:** 10.1038/s41415-025-8404-1

**Published:** 2025-04-11

**Authors:** Nicholas N. Longridge, Arindam Dutta, Kathryn Fox

**Affiliations:** 020066414914076351774 School of Dentistry, University of Liverpool, United Kingdom; 565398676800968699921 School of Dentistry, University of Cardiff, United Kingdom

## Abstract

This paper explores the current landscape and future direction of endodontic education, highlighting the crucial role of individual self-efficacy in both teachers and learners. Endodontic education is influenced by a wide range of factors and frameworks that enable students to develop the scientific knowledge and procedural skills that support evidence-based practice. Educators in endodontics require appropriate training to support students through pre-clinical training and into clinical practice. This paper discusses innovative educational strategies, such as the use of additively manufactured typodonts for realistic practice and complexity progression, as well as the integration of augmented and virtual reality (AR/VR) to enhance experiential learning. Additionally, we discuss the implications of artificial intelligence (AI) in personalising learning experiences and improving diagnostic skills. Through these discussions, we identify promising directions for future research and pedagogical innovation in endodontics.

## Key points

- Discusses the present status of endodontic teaching and the educational theory that supports it.
- Provides an overview of the current teaching strategies and tools in both pre-clinical simulated, and clinical teaching.
- Discusses the future educational innovations for enhanced experiential learning, such as AR/VR technologies and the integration of artificial intelligence into endodontic teaching.

## Introduction

The condition of the dental pulp and periapical tissues is an essential element of oral health and is directly linked to tooth retention, ultimately enhancing the quality of life.^1^ As such, endodontics remains a fundamental component of dental school curricula and clinical practice. While many of the principles in endodontics have remained consistent, the environment and media through which students learn and develop has evolved. This paper aims to discuss the present status and future direction of undergraduate and postgraduate endodontic learning. It will also discuss the current educational frameworks within endodontics, and the integration of new technologies to enhance both clinical confidence and self-efficacy.

## Development of the individual (teacher and learner)

As highlighted by Qualtrough,^2^ the teaching of endodontics can be influenced by a range of fixed frameworks and benchmarking documents, as well as variable factors such as geographic location, staff training, time allocated within the curriculum and access to educational resources or advanced equipment. Despite these variations, the common purpose of education and training in endodontics is to produce a practitioner who can demonstrate, on successful completion of the assessed programme, that they have met all the learning outcomes required for registration with their regulatory authority, either at undergraduate or postgraduate specialist level. Beyond this, educators and students must recognise the importance of continuing professional development, which extends well beyond the undergraduate degree. As such, a greater focus is now placed upon developing the individual, who according to the 2024 European Society of Endodontology (ESE) Undergraduate Curriculum Guidelines,^3^ should ‘not only be prepared but also feel prepared for their roles as dentists'. In order to feel prepared, students (and teachers) need to develop self-efficacy. Self-efficacy has been defined as ‘the strength of a person's belief in their ability to produce performances necessary for successful outcomes',^4^ also noting that if a person has the necessary motivation and appropriate skills, self-efficacy will be a major determinant in the actual performance delivered.

The aim of dental education is therefore not only to train students to have the knowledge and skills to be competent in undertaking endodontics, but also to build self-efficacy to enable capability (over a range of clinical contexts and increasing difficulty) across the individual's full practising career. This is essential in endodontics, where the full range of clinical complexities and case presentations is unlikely to be experienced by every student before graduation. Self-efficacy can be developed in four different ways:

1. Gaining mastery from experience. Therefore, it is very important that students receive adequate simulated and clinical endodontic experience, with appropriate increasing complexity.^5^ As most of the undergraduate training in the UK takes place within secondary care institutions, and those patients referred to dental hospitals, by their nature, require more complex treatment, it can be particularly difficult to provide students with the ideal amount of basic clinical endodontic experience, or with the appropriate increasing complexity of cases. This is where clinical outreach settings are desirable to provide access to patients requiring simple endodontic treatments. However, as highlighted in the 2024 ESE curriculum guidelines,^3^ the supervising clinical staff should ideally have ‘special knowledge, interest and self-efficacy' in endodontics, which may be more difficult to achieve in outreach settings.
2. Vicarious experience by assisting and observing peers. The benefits of this can be enhanced by the assistant being actively involved in the clinical care, such as by using video screens attached to the microscope, to facilitate discussion and learning.^6^
3. Verbal persuasion from a credible tutor enhances the student's belief that they possess the required capabilities to perform the task. Therefore, clinical tutors should be adequately trained in providing effective, future-focused feedback to their trainees.^7^
4. Physiological and emotional state. To facilitate the trainee's performance, attention should be paid to the environment in which the learning is taking place and the student's potential to deal with performance-induced stress.^8^ In addition, the emotional impact of giving and receiving difficult feedback should be recognised, and tutors should be equipped with strategies to deal with this.

## Frameworks for undergraduate teaching

A range of educational frameworks, resources and learning objectives have been developed to help support educators deliver endodontic education within the UK and Europe. These resources provide both a regulatory benchmark for registration where appropriate, along with learning outcomes for best practice in dental education and endodontic teaching:

- General Dental Council (GDC) - Safe practitioner framework^9^
- ESE - Undergraduate curriculum guidelines for endodontology^3^
- Association of Dental Education in Europe - Undergraduate curriculum framework^10^
- British Endodontic Society - A guide to good endodontic practice^11^
- GDC - Endodontics - specialty training curriculum.^12^

### Safe practitioner framework

In the UK, the GDC is transitioning from Preparing for practice to the Safe practitioner framework,^9^ replacing the terms ‘safe beginner' and ‘independent practitioner' with ‘safe practitioner'. This term encompasses not only the essential knowledge and skills for registered dental practitioners but also emphasises professional behaviours, interpersonal skills and self-management, including insight and reflection.

Becoming a safe practitioner in endodontics also requires accurate self-assessment, strong self-efficacy, and the ability to assess clinical complexity. With these, a practitioner can confidently perform tasks within their training, seek guidance when needed and, when appropriate, refer patients requiring specialist care.

### Learning outcomes

The ESE Undergraduate curriculum guidelines for endodontology^3^ identified clear learning outcomes for endodontics mapped as either ‘fundamental', ‘clinical' or ‘supporting clinical', based on the ADEE Undergraduate curriculum framework.^10^ In addition, higher education providers work to ensure graduates' experience aligns with the three tiers of competencies, which are: ‘be familiar with, have knowledge of, and be competent at'.^3^ Collectively, these documents provide a blueprint for all endodontic teaching, which help to deliver the scientific theory and evidence base for practice as well as development of clinical and professional behaviours to help perform safe endodontic treatment.

## The present status of endodontic teaching

### Pre-clinical - scientific knowledge and evidence base

Learning outcomes in endodontics are now commonly taught using a blended (hybrid) learning approach,^13^ in which face-to-face teaching is integrated with online synchronous and asynchronous teaching and learning.^14^ While this transition may have been accelerated by the COVID-19 pandemic,^15^ evidence to support blended learning approaches such as the flipped classroom has been available for the past two decades.^16^^,^^17^ Blended learning provides distinct advantages for both students and educators; these include improved accessibility, inclusion, flexibility, performance, satisfaction, and both reducing costs for providers and travel costs for students.^18^^,^^19^ However, educators must ensure adequate time for engagement with online resources and work to support deeper understanding and contextualisation of theory, during both face-to-face clinical teaching and simulated clinical cases.^20^

### Pre-clinical - procedural knowledge and behaviour-based learning

The dental head simulator or ‘phantom head' remains essential for development of posture, working position and psychomotor skills.^21^ Students spend substantial time within these environments working through a range of simulated tasks using a range of learning resources from endodontic Perspex blocks, 3D-printed or injection-moulded typodonts and, where available, extracted human teeth. In addition, a range of virtual or augmented reality (VR/AR) devices are now widely available for pre-clinical dental training.^22^ Haptic technology that provides tactile feedback to students has now become commonplace within dental schools^23^ and integration of full VR headsets for dental training is gaining traction.^24^ Table 1 outlines the advantages and disadvantages of each modality for pre-clinical training in endodontics.

**Table 1:** Advantages and disadvantages of each modality for pre-clinical training in endodontics

**Modality**	**Procedural stages**	**Advantages**	**Disadvantages**
Perspex blocks **Figure:** 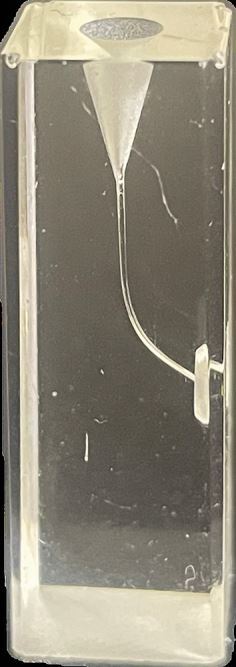	Canal negotiation Biomechanical preparation (De)obturation	Simple, benchtop design Cheap Good visualisation of obturations	Poor anatomical representation Poor compatibility with phantom head No pre- or post-endodontic build-up/restoration
Extracted human teeth **Figure:** 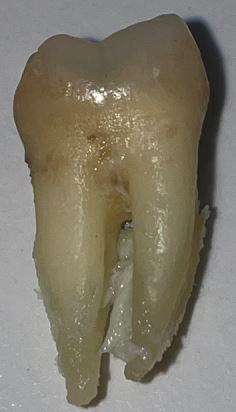	Full procedural stages including: Tooth and root assessment Access Canal identification Biomechanical preparation (De)obturation Post preparation Restoration	Fully anatomical including narrow canals/sclerosis Excellent tactile feedback from different dental tissues during access and preparation Real-world dentine colour changes for canal identification Low cost	Implications under the Human Tissues Act 2004 Limited standardisation across student cohorts Inability to control or predict potential challenges (eg pulp stones) Variable intra- and inter-year availability Radiographic interpretation required for identification of procedural errors and technical quality of obturations Cross-infection and disposal
Additively manufactured typodonts **Figure:** 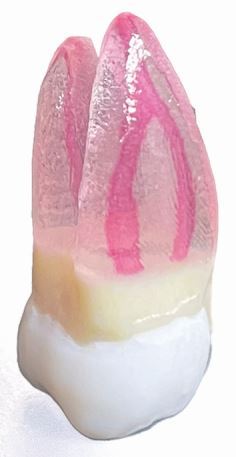	Full procedural stages including: Tooth, root and canal assessment Access Canal identification Biomechanical preparation (De)obturation Post preparation Restoration Surgical endodontics (with advanced model development)	Fully anatomical Good visualisation of procedural errors and obturations without radiographs (if translucent roots) Standardised design for each student and cohort which aids: Staff calibration for feedback Objective intra-year and inter-year comparison of performance and teaching practices Enables in-house production	Expensive if purchased High initial equipment costs and time-consuming if designed and produced in house Softer tactile feedback cf. enamel and dentine Artificial dentine colour changes for canal identification Acrylic/resin smear layer that can predispose to blockages, fractures and file separations Small canal diameters may be below available printer resolution (print-technology dependent) Difficulty removing support materials within root canal system
Augmented reality - haptics & force-feedback technology	Tooth and root assessment Access cavity preparation Canal preparation Surgical endodontics	Standardised and adaptable range of procedures Safe to use with no risk of sharps injuries or requirement for personal protective equipment Modules available for different specialties Real-time quantitative feedback (eg percentage tooth removal during access cavity) Unlimited repeats/procedures with low running costs Addition of further tasks/procedures is possible with software upgrades Gamification for students to set objective and measurable procedural targets	High initial purchase costs Further evidence is required to demonstrate procedural skills are fully translatable to clinical endodontic practice^25^ Requires ongoing technical software/hardware support
Virtual reality	Tooth, root and canal assessment Access cavity preparation Canal preparation Surgical endodontics	Fully immersive experience Unlimited repeats/procedures with low running costs Addition of further tasks/procedures is possible with software upgrades Gamification for students to set objective and measurable procedural targets	Requires combination with haptics to experience tactile feedback Nausea and dissociation Costs of headsets/equipment Limited realism including loss of tooth scale transitioning from VR to clinical practice

## Innovation in endodontic teaching

### Additively manufactured typodonts

Additive manufacturing of teeth and surgical models for endodontic teaching is evolving in higher education. Fully anatomical teeth can be created from cone-beam computed tomography (CBCT) or micro-computed tomography (μCT) scans, either in-house or via commercial providers.^26^ As 3D printer costs decrease and more dentists and technicians gain skills in additive manufacturing, this technology will become more widely used. These models standardise pre-clinical simulations and allow for structured progression in complexity, from simple procedures for undergraduates to complex cases and surgical endodontics in postgraduate training. Despite challenges to develop materials that mimic the hardness of biological tissues,^27^ initial research shows comparable technical outcomes in training.^28^ However, the correlation between typodont performance and patient technical outcomes is unclear.^29^ Thus, educators should view typodonts as a progressive learning tool rather than a replacement for proper support and feedback during clinical transition.

### AR/VR integration

The use of AR and haptics for access cavity preparation is well established in dental schools.^30^ However, the efficacy of using AR/VR to simulate complete endodontic treatments, such as canal preparation, obturation, or surgical endodontics, requires further research.^31^^,^^32^ While VR headsets are cheaper than haptic devices, they can lack convincing tactile feedback. Combining haptic technology with VR is likely to expand in the next decade, with more research needed to guide educators on when to integrate each into pre-clinical training.

### Artificial intelligence

All aspects of healthcare, including endodontics, have seen artificial intelligence (AI) applications emerge that will likely revolutionise clinical practice and teaching. AI has been implemented in electronic patient records systems, as well as diagnostic procedures such as radiographic assessment, complexity assessment and treatment prognostication.^33^^,^^34^ In education, generative and taught AI systems are constantly evolving to allow for simulated patient conversations, history taking and diagnostic journeys that will likely become common practice for the pre-clinical student over the next decade.^35^ In addition, educational platforms are available that allow for accurate transcription of lectures from which flashcards and quizzes can be automatically generated.

## Transitioning to clinical practice

Whether using simulation, VR, or in-person teaching, the curriculum should include ‘scaffolding' (providing a temporary support to allow a novice to gain skills) from tutors. This helps trainees progress through Vygotsky's ‘Zone of proximal development' (ie the gap between what a trainee can't do alone and what they can achieve with guidance),^36^ ultimately leading to independent practice. Scaffolding is especially crucial in the transition from undergraduate to postgraduate training.

During clinical training, staff self-efficacy and endodontic expertise are vital for providing effective feedback and support when complexities arise^37^ and consequently the staff-to-student ratio in clinical sessions must be carefully managed,^38^ with a recommended maximum number of four endodontic treatments per staff supervisor.^3^ Furthermore, higher education providers face challenges with increasing student numbers, limited patient availability, and a shortage of clinical supervisors,^14^ which may be worsened by the NHS long-term workforce targets.^39^ As it is unlikely that all graduates will have experienced the full range of endodontic treatments at the point of graduation,^40^ a supportive transition through Dental Foundation Training (DFT) is essential. In response to this, the British Endodontic Society has collaborated with NHS providers to provide ongoing support for DFT educational supervisors and graduates in endodontics.^41^

## Transitioning to postgraduate education

**Fig. 1:**
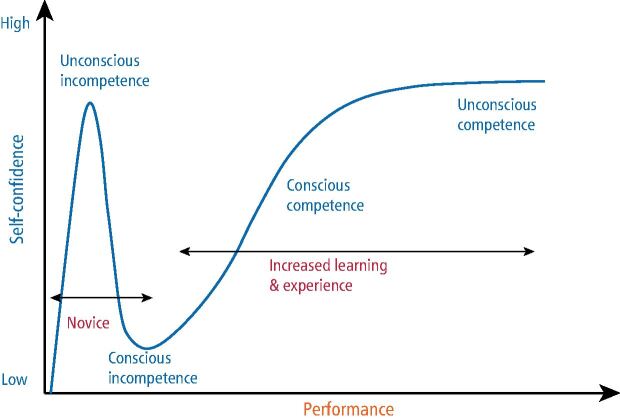
Dunning-Kruger effect

The value of the Educational transition document, which UK graduates take from university to DFT, depends partly on the student's self-assessment of their confidence. However, this can be influenced by the Dunning-Kruger effect (Fig. 1), which links task performance and self-awareness.^42^ Early-stage trainees may display overconfidence, or ‘unconscious incompetence'. As their experience grows, so does their understanding of potential consequences and self-awareness, leading to a drop in confidence (conscious incompetence). Only then, with proper support and personal success in more complex cases, trainees gradually move towards ‘conscious competence' and eventually ‘unconscious competence'. Candid discussions between the graduate and educational supervisor about the trainee's position on this curve are crucial. Notably, male students tend to report higher confidence than female students, without matching levels of competence,^43^^,^^44^ so educational supervisors should be trained to conduct supportive, coaching-style conversations to ascertain a trainee's actual capabilities.

## Assessment - authentic assessment

In both undergraduate and postgraduate education, ‘assessment of learning' and ‘assessment for learning' is crucial for developing effective practitioners. To foster self-awareness and self-efficacy, assessments should include reflection, self-assessment against clear criteria, and formative feedback that encourages growth and personal responsibility. Summative assessment of competence at basic or specialist levels is also necessary to meet awarding body standards and regulatory requirements. For fairness and equality, assessments must be reliable and valid.^45^ Many current clinical assessment methods are psychometrically analysed for reliability, but this can reduce authenticity in favour of objectivity. To achieve authentic assessment, higher education providers should use longitudinal assessments across various clinical contexts, complexities, and examiners to evaluate a student's true capability.^46^

## Postgraduate education in endodontics in the UK

Postgraduate education in endodontics in the UK offers several pathways for graduates to further develop, or even specialise.^47^ These choices are influenced by career goals, the desire to enhance clinical skills, and personal experiences during their initial training. Hospital-based specialty training, overseen by postgraduate Deaneries across the UK, allows students to pursue specialist training in restorative dentistry or endodontics through NHS and academic pathways. Academic positions are ideal for those aspiring to teach and/or engage in research. Training institutes are commonly accredited by the ESE against set standards, which requires at least 4,500 hours of training amongst other requirements.^48^

Upon completing hospital-based specialty training and passing membership exams from relevant Royal Colleges, students receive a Certificate of Completion of Specialist Training (CCST) from the GDC, enabling them to join the specialist list. Alternatively, there are self-funded, university-based postgraduate programmes lasting three years (or part-equivalent), which also follow the GDC's specialty curriculum.^12^ However, these latter programmes do not automatically qualify students for the specialist list; graduates must apply separately via the GDC's assessed portfolio process.

Specialty training in the UK usually takes place in secondary or tertiary healthcare settings, where advanced tools like dental operating microscopes, CBCT, thermal obturation units, ultrasonic devices and contemporary evidence-based dental materials are available. These facilities and UK referral pathways allow students to treat progressively more complex cases, which are often referred from primary care settings.^49^ In addition, digital technologies such as static and dynamic navigation are increasingly used for both surgical and non-surgical endodontic procedures.

Training follows the cognitive apprenticeship model, where trainees work closely with clinical supervisors and progress to more complex cases over time (scaffolding). Trainees are encouraged to reflect on their learning experiences, which helps tailor individual learning plans. This model accommodates students from various backgrounds, allowing them to move from competence towards proficiency and specialisation.^50^ Senior trainees often mentor junior colleagues, further enhancing their growth within this supportive environment.

Alongside the need for specialist endodontists, there is also a demand for dental practitioners with intermediate skills to manage presentations beyond the capacity of general dentists but not complex enough to require specialist intervention^51^ These ‘Tier 2' practitioners can acquire advanced skills through various training routes, including university-validated, or private, programmes. These allow dentists to develop skills while working, often within their own practice, enabling them to ‘learn as they earn'. Many such programmes are designed to allow enrolment, mentoring and coaching at different career points, providing flexibility and support that can span over several years.

Many general dental practitioners (GDPs), where they lack confidence^52^ or postgraduate qualifications,^53^ will refer patients needing endodontic care. To address this, these clinicians often pursue continuing professional development (CPD) as part of a personal development plan, which can involve online resources, short courses, or technological training. However, while CPD may improve knowledge and adoption of new technology,^54^ it may not necessarily enhance treatment outcomes.^55^ Recently, blended learning models have emerged, combining online materials with practical skill-building exercises, allowing practitioners to simulate complex procedures remotely,^56^ although further research is needed to evaluate the effectiveness of these methods across different endodontic treatments.

## Conclusions

This paper highlights that endodontic education is multifaceted. Ongoing technological developments require graduates to emerge with high self-efficacy that will support life-long learning across their full career. The integration of new educational technologies has enhanced delivery of theoretical and procedural knowledge, while mitigating the challenges of increasing student numbers with a concurrent decline in the availability of educational supervisors.

The recognition that endodontic cases range in complexity necessitates the training of clinicians with the full range of clinical competencies. This training extends beyond undergraduate education, DFT and into ongoing postgraduate education and development.

## Data Availability

No primary data are contained within this manuscript. Requests for further information can be submitted to the corresponding author.
